# A confined-etching strategy for intrinsic anisotropic surface wetting patterning

**DOI:** 10.1038/s41467-022-30832-4

**Published:** 2022-06-02

**Authors:** Rui Feng, Fei Song, Ying-Dan Zhang, Xiu-Li Wang, Yu-Zhong Wang

**Affiliations:** The Collaborative Innovation Center for Eco-Friendly and Fire-Safety Polymeric Materials (MoE), National Engineering Laboratory of Eco-Friendly Polymeric Materials (Sichuan), State Key Laboratory of Polymer Materials Engineering, College of Chemistry, Sichuan University, Chengdu, 610064 China

**Keywords:** Surface chemistry, Information storage, Surfaces, interfaces and thin films, Wetting

## Abstract

Anisotropic functional patterned surfaces have shown significant applications in microfluidics, biomedicine and optoelectronics. However, surface patterning relies heavily on high-end apparatuses and expensive moulds/masks and photoresists. Decomposition behaviors of polymers have been widely studied in material science, but as-created chemical and physical structural changes have been rarely considered as an opportunity for wettability manipulation. Here, a facile mask-free confined-etching strategy is reported for intrinsic wettable surface patterning. With printing technology, the surface wetting state is regulated, enabling the chemical etching of setting locations and efficient fabrication of complex patterns. Notably, the created anisotropic patterns can be used for realizing water-responsive information storage and encryption as well as fabricating flexible electrodes. Featuring advantages of simple operation and economic friendliness, this patterning approach brings a bright prospect in developing functional materials with versatile applications.

## Abstract (web summary)

Anisotropic functional patterned surfaces have shown significant applications in microfluidics, biomedicine, and optoelectronics. Here, authors demonstrate a fast and mask-free etching method for accurate surface patterning by confined decomposition, enabling the efficient fabrication of complex patterns.

## Introduction

Natural patterns, always, have the power to catch eye and intrigue mind. People show enthusiasm to find reasons for the natural patterns seen in creatures of the land, sky and sea (spots of cheetah, feathers of parrot, sucker-arms of octopus, etc.). Therein, some patterns endow anisotropic wettability as survival essentials to some organisms (desert beetle, lichen and rice leaf, for instance)^1–3^. Inspired by these fantastic phenomena, artificial patterns with anisotropic wettability have been fabricated to realize directional adhesion and liquid manipulation, showing great potential in both fundamental and industrial applications. Surface chemical composition and physical morphology are two key factors that determine surface wettability^4–6^. Generally, the construction of a single anisotropic physical morphology can only achieve axial anisotropic wetting^7–9^. In contrast, anisotropic chemical composition or hybrid chemical/physical anisotropy enables an obvious hydrophobic/hydrophilic boundary between the pattern and back regions, showing promising applications in cell arrays^10–12^, biosensors^13,14^, fog harvesting^15,16^, printing technology^17,18^, droplet manipulation^19,20^ and optoelectronics^21^. To date, great efforts have been made on microfabrication of asymmetric wettable patterns, including photo-grafting^22–24^, electro-chemical etching^25,26^, plasma treatment^27–30^, photo-lithography^31–34^, micro-milling^35,36^ and laser ablation^37,38^. Despite the construction of precise patterns using these methods, the patterning relies seriously on expensive apparatuses (e.g., femtosecond laser, lithography machine), complex molds/masks, and sometimes complicated and time-consuming multiple-step synthetic processes, becoming the main limitations to wide industrial promotion. In comparison, mask-free ink-jet printing and depositing techniques have been explored for surface wetting patterning in recent years^8,17,39–44^, showing high potential for scalable production due to the low cost and pattern richness. Regarding the techniques, inks must adhere consistently to solid surfaces; hence, the coffee ring effect and interfacial forces that seriously affect the precision and stability of patterns^45–47^ are required to consider significantly. Moreover, exposure of the ink track is always difficult to avoid. Therefore, in principle, it is almost impossible to realize information/pattern encryption and reading in terms of wetting/nonwetting manipulation. Compared to the extrinsic way to achieve anisotropic wetting, if intrinsic patterns can be depicted directly by chemical etching of a solid matrix, high stability and even invisibility of patterns will be enabled.

Decompositions of polymer materials have been widely investigated for several decades; however, relevant chemical and physical structural changes are mostly considered from macroscopic and integrated perspectives of materials, and it is still unknown whether confined and local molecular decompositions can be used for intrinsic surface patterning.

**Fig. 1:**
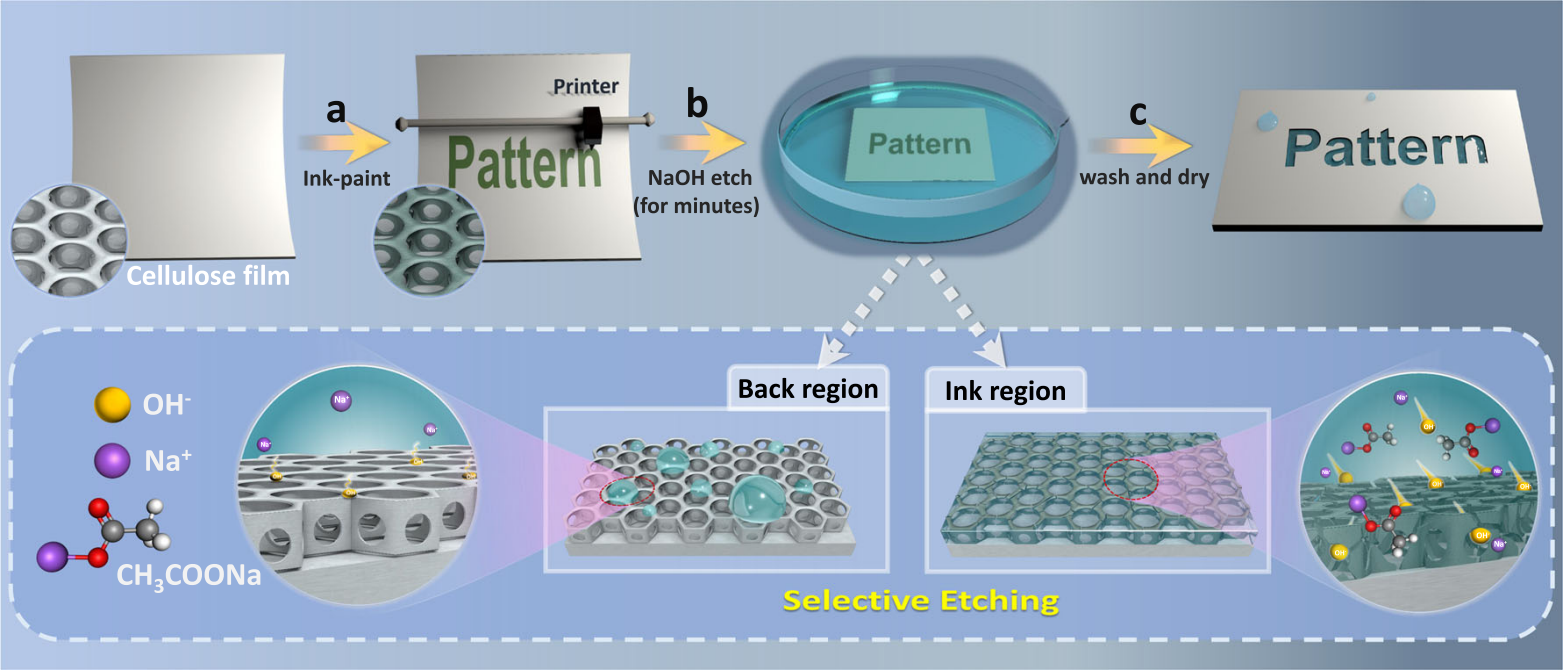
Fabrication strategy of accurate patterns with anisotropic wettability. **a** Painting ink on microporous cellulose triacetate film to temporarily construct anisotropic water penetration ability. **b** Confined surface etching upon the treatment with aqueous NaOH solutions. **c** Washing the patterned surface to remove excess NaOH and drying for permanent patterns.

Here, we report a facile and fast mask-free etching method for accurate surface patterning by controlling the confined decomposition of material surfaces (Fig. 1). With a common printing technique and subsequent location-confined chemical etching, intrinsic, complex and accurate patterns with a resolution of 200 μm are fabricated (such as QR code). In particular, an etching processing window is established for information storage and encryption. The as-designed patterns/information can be hidden in the film but read after exposure to external stimuli (water/moisture, for instance). Due to the selective growth of metal conductors in wettable patterns, flexible electronics with desired styles can also be fabricated. Such a mask-free and simple method shows great potential in the mass production of accurate functional patterns from a variety of polymeric materials.

## Results

### Tunable morphology and wettability

**Fig. 2:**
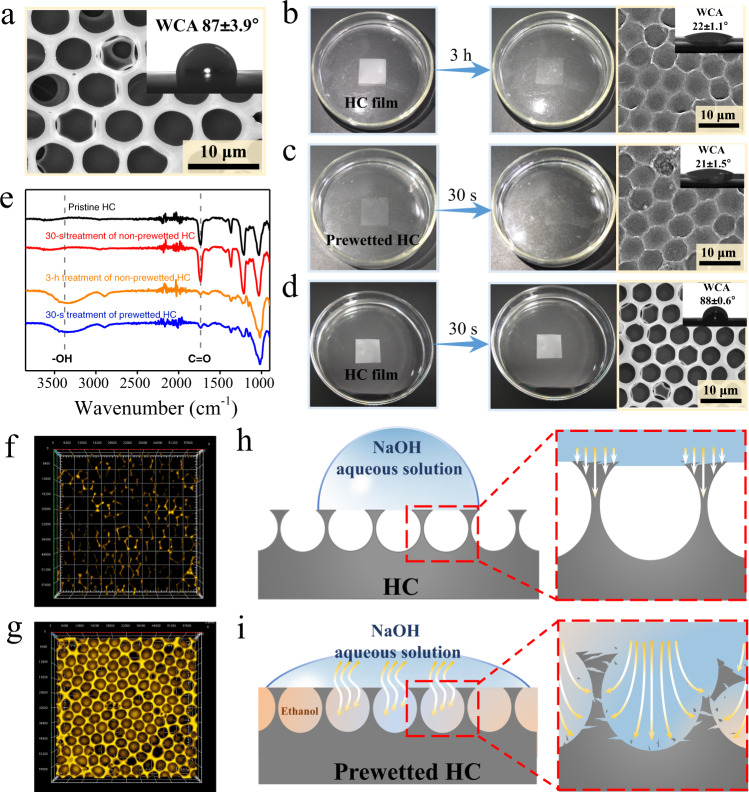
Tunable NaOH-etching. **a** SEM image and water contact angle (WCA) of the original HC surface. **b** SEM image and WCA of the HC surface after 3-h NaOH-etching. SEM image and WCA of prewetted HC (**c**) and non-prewetted HC (**d**) surfaces after 30-s NaOH-etching. **e** ATR-FTIR spectra of HC film and the etched samples. LSCM fluorescence images of HC (**f**) and prewetted HC (**g**) surfaces after 30-s immersion in rhodamine B-stained water. Schematic illustration of the etching process of HC (**h**) and prewetted HC (**i**) films. Source data are provided as a Source Data file.

At first, a microporous cellulose triacetate (CTA) film is prepared according to our previous work by a breath figure method^9,48^. Figure 1a and Supplementary Fig. 1a show typical top and cross-section SEM images of the as-prepared microporous film, respectively. The surface shows an obvious 3D honeycomb-like (HC) microporous structure with a thin top monolayer, and the water contact angle (WCA) is 87 ± 3.9° (5 μl of droplet volume). After immersing the HC film in 5 M NaOH aqueous solution for 3 h, the resulting film displays obviously increased transparency (Fig. 2b). From the SEM images of the treated film (Fig. 2b and Supplementary Fig. 1b), the top monolayer disappears, and rough structures appear in the exposed micropores. Meanwhile, the WCA of the surface significantly decreases to 22°. More interestingly, if prewetting the HC film with ethanol in advance, only 30-s alkaline treatment can also achieve the same effect (Fig. 2c). As a comparison, a non-prewetted HC film that is merely treated in 5 M NaOH aqueous solution for 30 s shows almost unchanged microstructure, opacity and WCA (Fig. 2d). As shown in Fig. 2e, compared to the pristine HC film, both etched samples (3-h treatment of non-prewetted HC and 30-s treatment of prewetted HC) display severely weakened absorbance at ~1737 cm^−1^ (C=O) and enhanced absorbance at ~3331 cm^−1^ (-OH), indicating that the formation of etched porous structures is due to the base-catalytic deacetylation of CTA^49–52^. Meanwhile, the changed microstructure and exposed -OH group contribute to the improved hydrophilicity. In comparison, the almost unchanged peaks of C=O and -OH groups suggest no deacetylation in the case of non-prewetting but only 30-s alkaline treatment.

To understand why the pretreatment with ethanol is favorable to the surface etching, the surface wettability as well as the liquid penetration into the solid matrix is investigated. Despite the WCA of only 87 ± 3.9°, the HC surface still shows well water repellency with free droplet sliding (Supplementary Fig. 2a and Supplementary Movie 1, 30 μl of droplet volume). As a result, the water repellency causes the hindrance of the aqueous NaOH solution into micropores but the location mainly at intervals, that is, relatively weak and poorly distributed fluorescence is observed after the immersion in rhodamine B-containing water (Fig. 2f). In contrast, ethanol can rapidly wet the HC surface (Supplementary Fig. 2b), resulting in the fast entry of NaOH aqueous solution into the cavity. Correspondingly, strong fluorescence from rhodamine B is observed in the micropores after the same immersion treatment (Fig. 2g). Therefore, the results indicate that the ease penetration of the NaOH aqueous solution after ethanol pretreatment enables faster etching toward a hydrophilic HC surface (Fig. 2h, i).

### Construction of wettable patterns

**Fig. 3:**
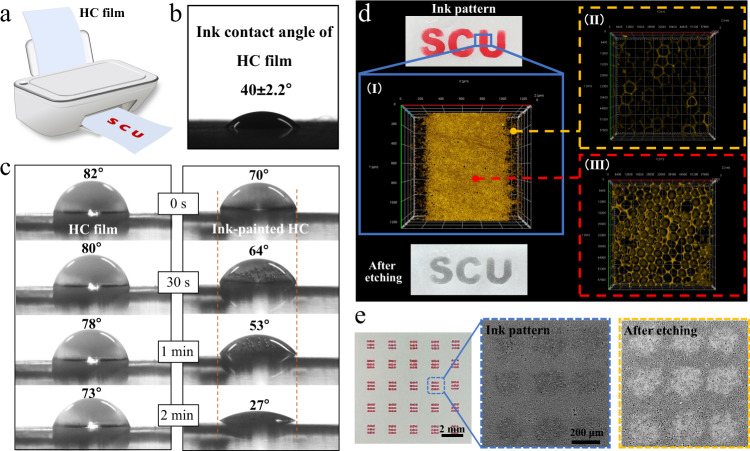
Ink-jet-printing-assisted patterning. **a** Illustration of ink-painting on HC surface via an ink-jet printer. **b** Contact angle of ink on HC surface. **c** Water wetting states of HC and ink-painted HC surfaces. **d** Ink pattern on HC film and the etched sample with “SCU” pattern. Partial LSCM fluorescence images of ink-painted HC after 2-min immersion in rhodamine B-stained water (I: box selected region, II: unpainted region, III: painted region). **e** Patterned surface with micro-dot arrays.

Because of the tunable etching and water wettability, an ink-jet printer is used to construct wettable patterns on non-wettable HC surface (Fig. 3a). Considering the fast evaporation and instability of ethanol as well as the difficulty in accurate patterning (Supplementary Fig. 3), a commercial ink containing water-soluble dyestuff and high-boiling-point alcohols (isopropanol, glycerol, etc.) is used instead. Thus, the ink can easily wet the porous structure of HC (Fig. 3b), and the wetted location shows partly covered pores and changed elemental components (Supplementary Fig. 4a, b). The abundant -OH group of the ink (Supplementary Fig. 4c) enables the increased hydrophilicity of the painted HC surface (Fig. 3c); that is, the water droplet is stable with relatively unchanged WCA for pristine HC but wets into the ink-painted HC surface with gradually decreased WCA and an unmoved three-phase line. Additionally, the rough microporous structures can stably maintain the shape of the painted ink, which is a key point ensuring the accuracy of the patterns; while on a smooth CTA surface, the ink tends to contract and deform gradually with time (Supplementary Fig. 5). From the dynamic etching process (detailed discussion provided in the Supplementary Information, Supplementary Fig. 6 and Supplementary Movie 2), the entry of aqueous NaOH solution into ink-painted micropores is observed directly, and a critical state referring to the disappearance of 3D micropores is determined. Accordingly, the aqueous NaOH solution can be designed to selectively enter the ink-painted domains, creating patterns after the surface etching (Fig. 3d). More importantly, with the ink-jet printing technique, precise, complex and tiny dot-like arrays with a resolution of 200 μm can be fabricated (Fig. 3e).

**Fig. 4:**
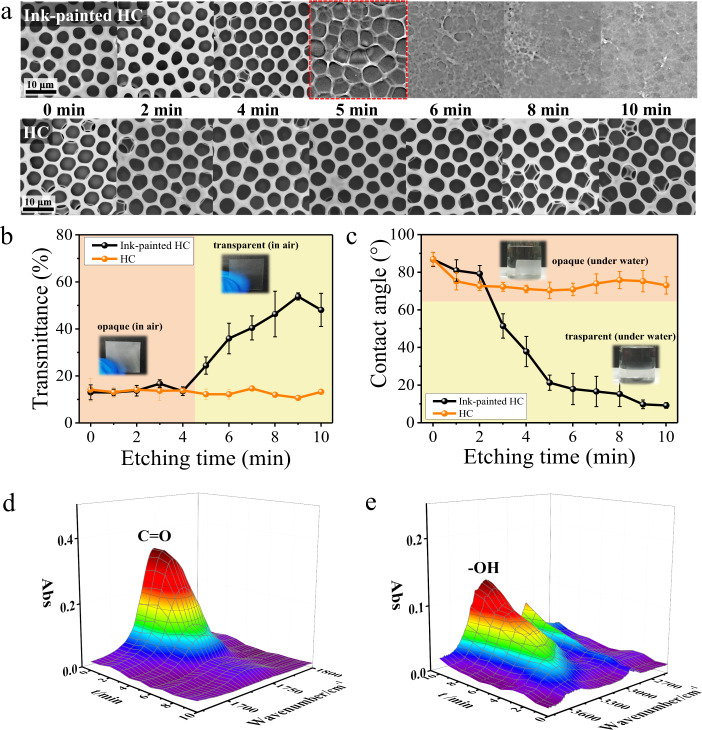
Controllable morphology and wettability. **a** SEM images of HC and ink-painted HC within 10-min etching. **b** Transparency of HC and ink-painted HC within 10-min etching (inserted are in-air photos of ink-painted HC before and after 5-min etching). **c** WCAs of HC and ink-painted HC within 10-min etching (inserted are under water photos of ink-painted HC before and after 3-min etching). ATR-FTIR spectra of C=O (**d**) and -OH (**e**) groups for ink-painted HC within 10-min NaOH-treatment. A 1 M NaOH aqueous solution was used here. The error bars in **b**, **c** show standard deviations based on three independent measurements. Source data are provided as a Source Data file.

Based on the effect of NaOH concentration on the etching (Supplementary Fig. 8), 1 M NaOH was fixed for further investigations. Regarding the dynamic treatment process, structural changes of the HC with and without ink-painting are recorded after the etching for different periods (Fig. 4a). With increased etching time, non-painted HC remains intact 3D microporous structures, while the top monolayer disappears and rough structures appear for ink-painted HC after 5-min etching. For ink-painted HC, at the initial time of 0–4 min, the 3D micropores remain well, and the film always remains opaque in air, while it becomes obviously transparent once the etching time reaches 5 min because of the broken micropores (Fig. 4b). In general, strong scattering of light occurs in the presence of a porous surface, resulting in opacity; if the pores are damaged or infused with transparent liquids, an increasing transparency is realized due to the weakened light scattering (Supplementary Fig. 9)^53,54^. Meanwhile, ink-painted HC shows continuously improved wettability with gradually decreased WCA within 10-min etching (Fig. 4c). It is worth noting that, the ink-painted HC film becomes transparent under water after 3-min etching. It can be concluded that, when the etching time is 3–4 min, the transparency change of the film under water is only caused by improved wettability and strong water infusion into the cavity of 3D micropores; while after 5-min etching, the transparency change also results from the damage of the pores. Furthermore, the continuous weakened infrared absorbance of the C=O group (Fig. 4d) and enhanced infrared absorbance of the -OH group (Fig. 4e) indicate the continuous deacetylation of the ink-painted HC within 10-min. In sharp contrast to ink-painted HC, non-painted HC remains unchanged transparency and wettability because of almost no deacetylation within 10 min. Additionally, the drying state of the ink-painted patterns is also found to just affect the surface wettability and etching rate, due to gradually weakened surface wettability with storage time and drying temperature, and the etching can be also conducted normally (Supplementary Figs. 10–14). Considering the preparation efficiency and reliability, the etching treatment is conducted constantly within 1-h storage for following investigations. For simplicity, according to the etching time, the resulting morphology, wettability, and transparency after etching are summarized in Supplementary Table 1.

### Information storage and encryption

**Fig. 5:**
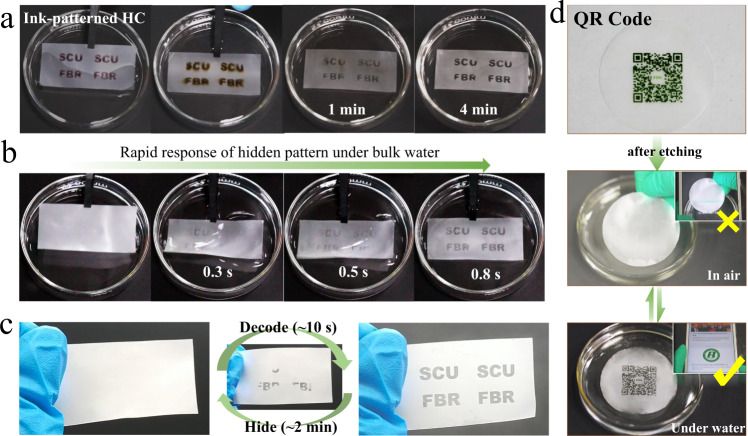
Information storage and encryption. Preparation (**a**) and rapid water response (**b**) of the hidden pattern “SCU FBR”. **c** Reversible decryption of the hidden information by water mist. **d** A QR code ink-printed on HC film before NaOH-treatment and the reading process of the hidden QR code after NaOH-treatment.

At present, stimulus-responsive encryption materials with convenient information hiding and decryption have gained increasing attention^55–57^. According to Supplementary Table 1, when treating an ink-patterned HC in 1 M NaOH aqueous solution for 3–4 min, the as-prepared film (including pattern and background regions) is opaque and the pattern is invisible at dry state, while in water, only the pattern region becomes wettable and transparent; this suggests a special time-window which is suitable for the fabrication of hidden patterns. As shown in Fig. 5a, taking advantage of such a time-window, a hidden pattern with “SCU FBR” characters is created. It should be noted that, from the surface and cross-section SEM images of a patterned HC surface (Supplementary Fig. 15), 3D micropores without obvious morphological differences are detected for the background and pattern regions. This further suggests that the etching occurs in terms of the chemical decomposition rather than physical microstructural changes; thus, the non-dominant etching cannot be felt by touching. More importantly, although the entry of the alkaline solution into the ink-painted area is accompanied with the ink dissolution, the etching precision is not reduced because of the high etching rate. Notably, the hidden pattern becomes visible immediately upon the contact with water, showing a fast water response (Fig. 5b and Supplementary Movie 3). In addition to bulk water, the hidden information can also be read with water mist (Fig. 5c and Supplementary Movie 4), although a longer decoding time is required. To further control the decoding time, the flow rate of water mist is adjusted. As shown in Supplementary Fig. 16, as the flow rate increases from 80 to 200 mL/h, the decoding time decreases from 390 s to below 10 s. For encryption, steady hiding of codes in ambient surroundings is vital, so we further investigate whether code leakage occurs in highly humid air. As shown in Supplementary Fig. 17 and Movie 5, even at 100% RH (25 °C), the hidden information remains invisible. This suggests that the functional patterned surface shows only response to liquid water (e.g., bulk water and water mist) but high stability in air, guaranteeing the encryption application. Moreover, in common humid air, the code can be read by the cooling of the film, as a result of condensations of vaporous water into liquid water. Once cooling stops, the visible patterns become invisible rapidly again, and such hiding/revealing performance can be repeated for at least ten runs (Supplementary Fig. 18). Based on the high patterning precision, a complex QR code is also successfully created with the ink-jet printing and subsequent etching method (Fig. 5d), which appears quickly under water and can be read accurately (Supplementary Movie 6).

### Fabrication of flexible electronics

**Fig. 6:**
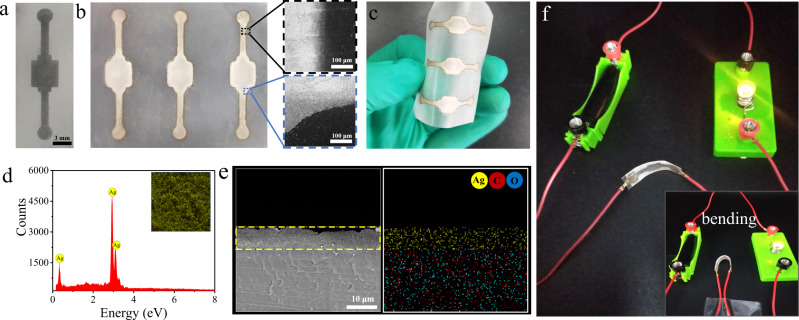
Fabrication of flexible electronics. **a** Digital image of the patterned EHC. **b** Digital and SEM images of Ag electrodes. **c** Photograph of the bent Ag electrodes. **d** EDS spectra of the Ag electrode (inserted are element mapping images). **e** Cross-section SEM image of the Ag electrode and corresponding element mapping image. **f** Photograph of a conductive loop equipped with the Ag electrode. Source data are provided as a Source Data file.

Flexible electronics have been applied to various fields, such as displays, sensors and medical appliances. Ag nanomaterials are commonly used to fabricate flexible electronics due to their high conductivity and mechanical flexibility^58–60^. Here, Ag nano-particles (NPs) are prepared in situ by conducting the silver mirror reaction. As shown in Supplementary Fig. 19a–c, Ag NPs can form on the surface of smooth CTA, HC as well as the etched HC (EHC, 10-min treatment by 1 M NaOH aqueous solution) after dripping a mixture of glucose aqueous solution and Tollens’ reagent. However, only on the EHC, a quite close and continuous Ag layer is observed, benefitting from the appropriate hydrophilicity and roughness. For CTA film, its surface is too smooth to retain Ag NPs; for HC surface, due to the water repellency, Ag NPs can only grow randomly outside of pores, resulting in an inconsecutive Ag layer. As results, the electrical conductivity of the EHC-Ag film is as high as 63.9 × 10^6^ S cm^−1^, which is much greater than those of CTA-Ag and HC-Ag (Supplementary Fig. 19d). Moreover, the EHC-Ag film can undergo repeating bending with minimal changes in the relative resistance and conductivity (Supplementary Fig. 19e). Therefore, the prepared patterned EHC surface can be directly used to construct flexible electronics. The patterned EHC surface and resulting Ag electrode are shown in Fig. 6a, b, respectively. The bound of the Ag electrode can be clearly observed from the SEM images (Fig. 6b), and the Ag electrode has high flexibility (Fig. 6c). The EDS spectra and element mapping images of the Ag electrode clearly show the formation of a dense Ag layer (Fig. 6d, e). Moreover, we also demonstrated that the lamp in the conductive loop could work very well before and after bending the Ag electrode (Fig. 5f and Supplementary Movie 7).

## Discussion

Based on the deacetylation of the HC surface in NaOH aqueous solution, intrinsic wettable patterns are fabricated easily and rapidly by an ink-jet-printing-assisted surface etching method. The morphology and wettability can be controlled exactly by adjusting the NaOH concentration and etching time. On the basis of printing technology, complex patterns are depicted accurately on the surface. With selective wettability, pattern information can be stored and encrypted on the cellulose film; upon exposure to external stimuli, such as water, encryption keys can be read. Moreover, such a method can also be used to prepare functional materials, flexible electronics for instance. The as-prepared Ag electrode presents high electrical conductivity (63.9 × 10^6^ S cm^−1^) and bending-deformation resistance. This work provides a scalable surface wettable patterning strategy, but three requirements are required for material substrates: (1) chemical reactions/treatments (including chemical degradation, decomposition, and even conjugation) can be conducted for substrate surfaces; (2) the substrate surfaces can be written with inks and can well maintain ink patterns; (3) the original region and ink-printed region have different wettabilities to etching agents, enabling confined-etching treatments. As long as any substrates satisfy the requirements, patterning is possible on their surfaces with this strategy. In addition, the ink in this work is water-soluble to allow the fast entry of aqueous NaOH solution into micropores. In future, more inks (oil-borne, for instance) are expected to be found and used for (super)hydrophobic substrates. We believe this mask-free and simple strategy can be applied for more material surfaces, bringing more opportunities for wide applications.

## Methods

### Materials

CTA was provided by Push Acetati Co., Ltd. (Sichuan, China). Dichloromethane, ethanol, sodium hydroxide (NaOH), rhodamine B and methylene blue were purchased from KeLong Co., Ltd. (Chengdu, China). Ink (HP 803) was purchased from Hewlett-Packard Co. Ltd. (America). All the materials were used without any further purification.

### Fabrication of HC film and wettable patterns

A 4 wt% CTA solution was first prepared in chloroform and then deposited in a glass culture dish (*d* ~ 75 mm). At 0.8 L/min flow rate of humid air and 25 °C, HC film was obtained as the evaporation of chloroform (~2 h). The wettable patterns were prepared by painting ink patterns on the HC surface with an ink-jet printer (HP DJ 1110) and then etching with 1 M aqueous NaOH solution for 3–10 min, followed by washing with deionized water to remove excess NaOH and then drying before use. Unless particular illustration, all the freshly ink-painted samples were etched within 1-h storage at 25 °C.

### Fabrication of flexible Ag electrode

To fabricate a flexible Ag electrode, a fresh mixture of 0.5 mL glucose aqueous solution (10 wt%) and 1 mL Tollens’ reagent (2 wt% AgNO_3_) was dripped on the pre-prepared EHC surface. After the mixture spread on pattern regions, the reaction was performed at 60 °C for 3 min, followed by washing with deionized water to remove the residual and then drying before use.

### Characterization

Morphological investigation was conducted by using a scanning electron microscope (SEM, Phenom ProX, Netherlands) at 10 KV. Element composition and distribution were determined by using an energy-dispersive X-ray spectrometer (EDS, Phenom ProX, Netherlands) at 15 KV. Before SEM and EDS tests, the surfaces were sprayed with gold for 60 s. An optical contact angle meter (JC200D2H, Zhongchen digital equipment Co. Ltd, China) was used to measure contact and sliding angles. Fourier transformed infrared spectroscopy was conducted on an infrared spectrometer (Nicolet 6700, Thermo Scientific, America). 3D fluorescence distributions were determined using laser scanning confocal microscopy (LSCM, LSM800, Carl Zeiss AG, Germany) under an excitation wavelength of 485 nm. Transmittance of samples was tested with an ultraviolet–visible spectrophotometer (Cary 50, Agilent, America). An optical microscope equipped with an electronic camera (CSW-3230A, Keshiwei, Chain) was used to record the dynamic etching process of samples. Conductivity and resistance were measured using a resistivity meter with a four-point probe (ST2258C, Suzhou Jingge Electronics Co., Ltd., China).

### Statistics and reproducibility

All data are expressed as the mean ± standard deviation and represent a minimum of three independent experiments. Details of statistical testing can be found in the figure legends and in the Source data file. Statistical analyses were carried out using Microsoft Excel. All SEM images, LSCM fluorescence images and digital photos shown are representative images selected from at least three independent experiments all showing similar results.

## Supplementary information

Supplementary Information

Description of Additional Supplementary Files

Supplementary Movie 1

Supplementary Movie 2

Supplementary Movie 3

Supplementary Movie 4

Supplementary Movie 5

Supplementary Movie 6

Supplementary Movie 7

## Data Availability

All data supporting the findings of this study are available within the article, as well as the Supplementary Information file, or available from the corresponding authors upon reasonable request. Source data are provided with this paper.

## Source data

Source Data
