# Supplementary material for: A confined-etching strategy for intrinsic anisotropic surface wetting patterning
Source: Nat Commun. 2022 Jun 2;13:3078. doi: 10.1038/s41467-022-30832-4 (PMC9163165; doi:10.1038/s41467-022-30832-4)
Supplement: Supplementary file 1 — Supplementary Information [file 41467_2022_30832_MOESM1_ESM.pdf]

Supplementary Information

A Confined-Etching Strategy for Intrinsic Anisotropic Surface Wetting Patterning

Rui Feng, Fei Song*, Ying-Dan Zhang, Xiu-Li Wang, Yu-Zhong Wang*

The Collaborative Innovation Center for Eco-Friendly and Fire-Safety Polymeric Materials (MoE), National Engineering Laboratory of Eco-Friendly Polymeric Materials (Sichuan), State Key Laboratory of Polymer Materials Engineering, College of Chemistry, Sichuan University, Chengdu 610064, China

** Corresponding authors.*

E-mail: songfei520@gmail.com (F. Song); polymers@vip.126.com (Y.Z. Wang)

Supplementary Figures

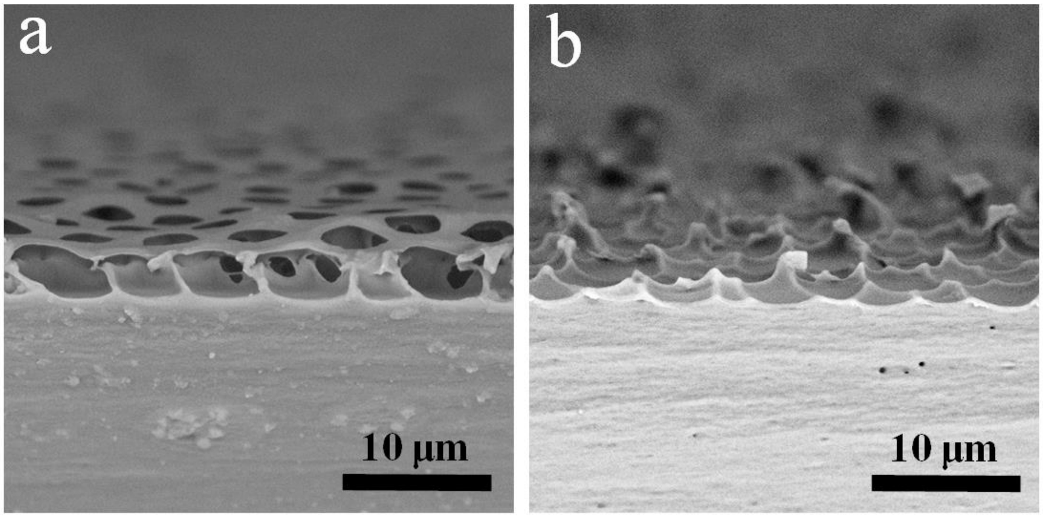

Supplementary Figure 1. Etching of micropores. a Cross-section SEM image of original HC surface. **b** cross-section SEM image of the HC surface after 3-h etching by 5 M NaOH aqueous solution.

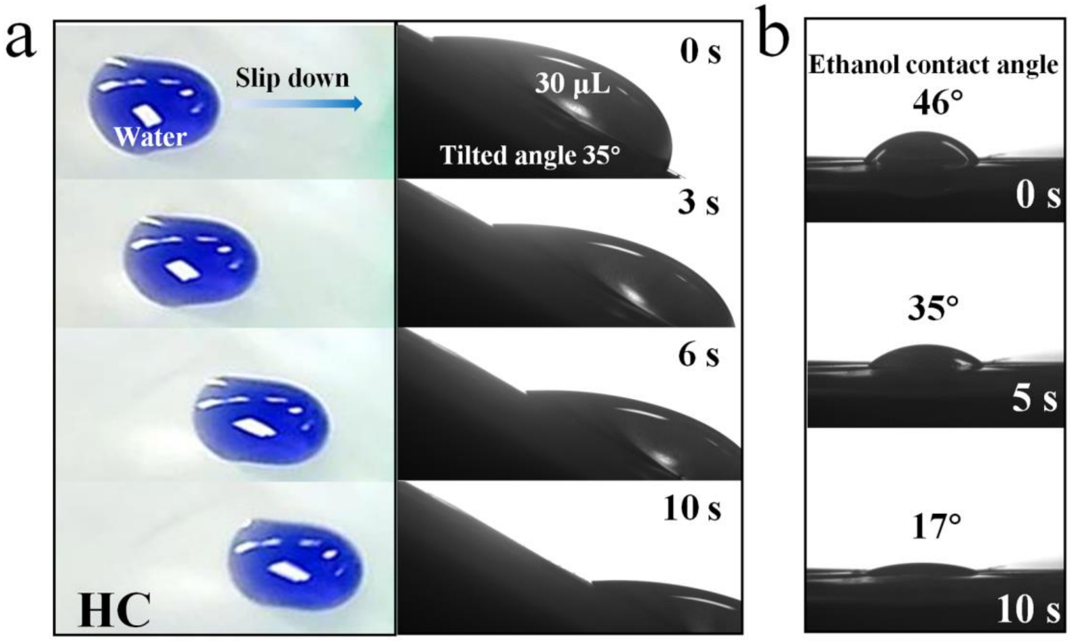

Supplementary Figure 2. Wettability of HC surface. a Free water droplet sliding on HC film and corresponding water sliding angle. **b** Ethanol contact angle of the HC surface.

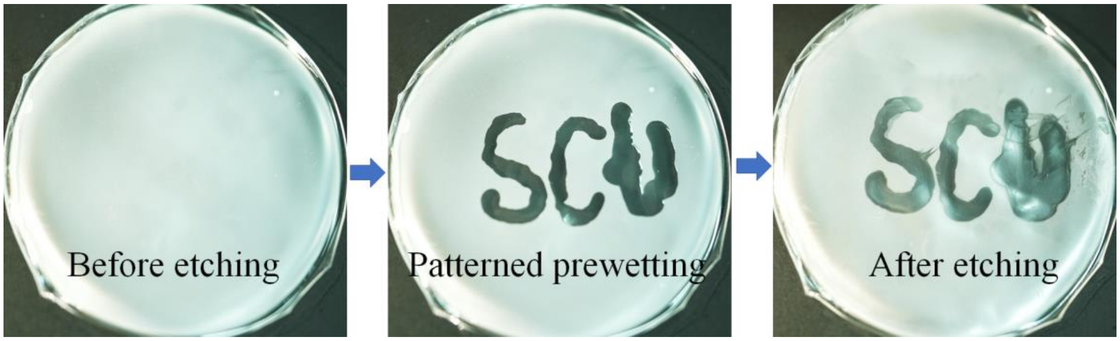

Supplementary Figure 3. Patterning etching. Preparation of a simple pattern by drawing ethanol on HC and then immersing the surface in 5 M NaOH aqueous solution.

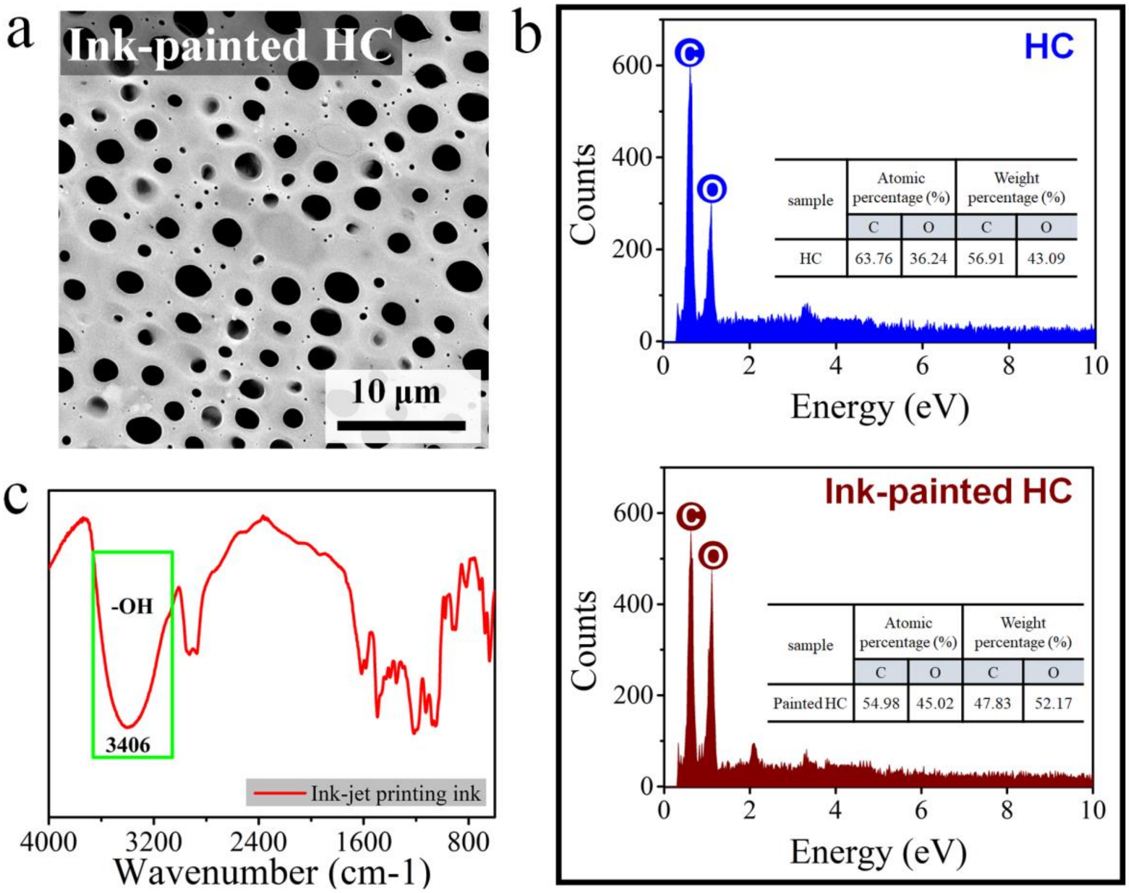

Supplementary Figure 4. Surface composition and morphology of ink-painted HC. **a** SEM image of the ink-painted HC surface. **b** EDS spectra of HC and ink-painted HC surfaces (inserted are the atomic and weight percentages). **c** ATR-FTIR spectra of dried commercial ink. Source data are provided as a Source Data file.

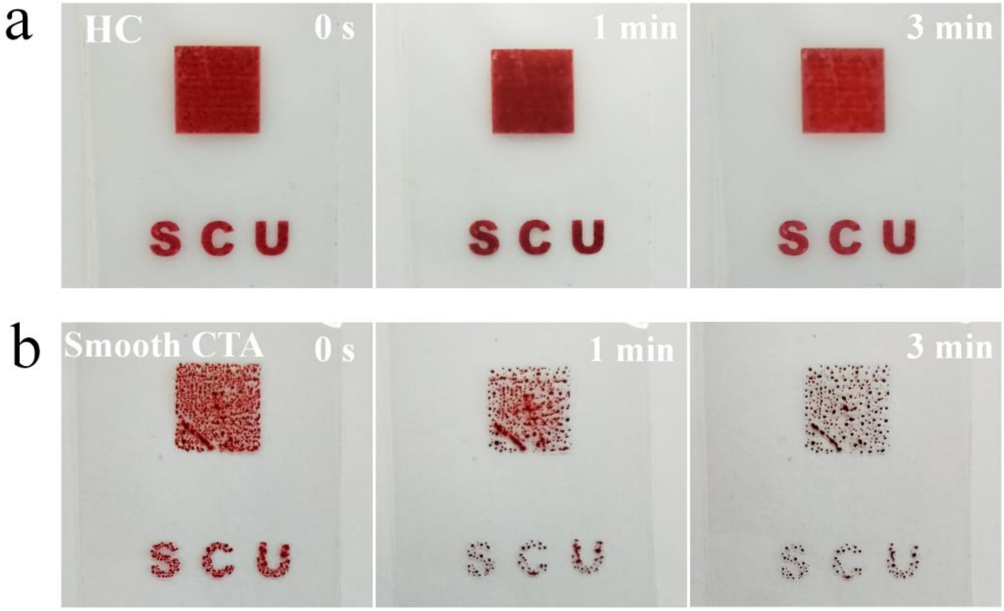

Supplementary Figure 5. Ink-pattern evolution. a Stably maintained Ink-pattern on HC surface. **b** Gradually deformed Ink-pattern on smooth CTA surface with time.

Supplementary Note 1. Dynamic etching process of ink-painted HC

The dynamic etching process of the ink-painted HC is monitored with optical microscopy (Supplementary Fig. 6 and Movie 2), where various stages can be seen. At first (stage I-II), many microbubbles are seen as the entry of the aqueous NaOH solution into the film micropores. With the complete emission of the trapped air from the micropores, the microbubbles disappear (stage II-IV). As the etching proceeds, the 3D microporous morphology disappears suddenly once the top layer is completely etched (stage V). This state is named critical state, and the corresponding time point is defined as the critical etching time. With further etching, obvious damages to the porous structure occur (stage VI), so the etching is terminated at stage V for controlled surface patterning. Meanwhile, no bubbles are observed for the smooth CTA surface during the same etching treatment (Supplementary Fig. 7a), although a decrease in the WCA is detected (Supplementary Fig. 7b). Therefore, it can be deduced that the formation of microbubbles is caused by the squeezing of the trapped air in micropores as water enters.

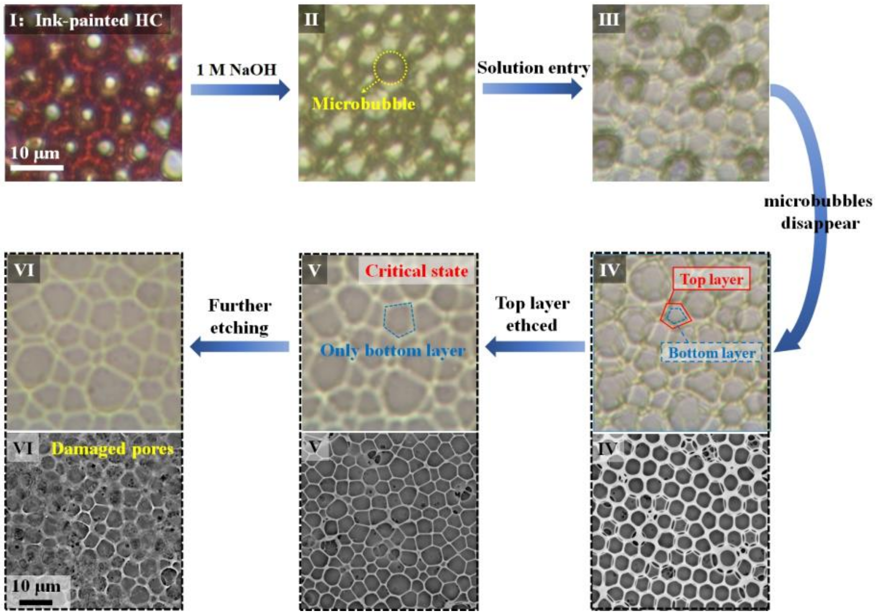

Supplementary Figure 6. Dynamic etching process of ink-painted HC. Optical micrographs of ink-painted HC during the dynamic etching process (stage I~VI). Additional SEM images reflecting the surface morphology are supplied for stages IV, V and VI.

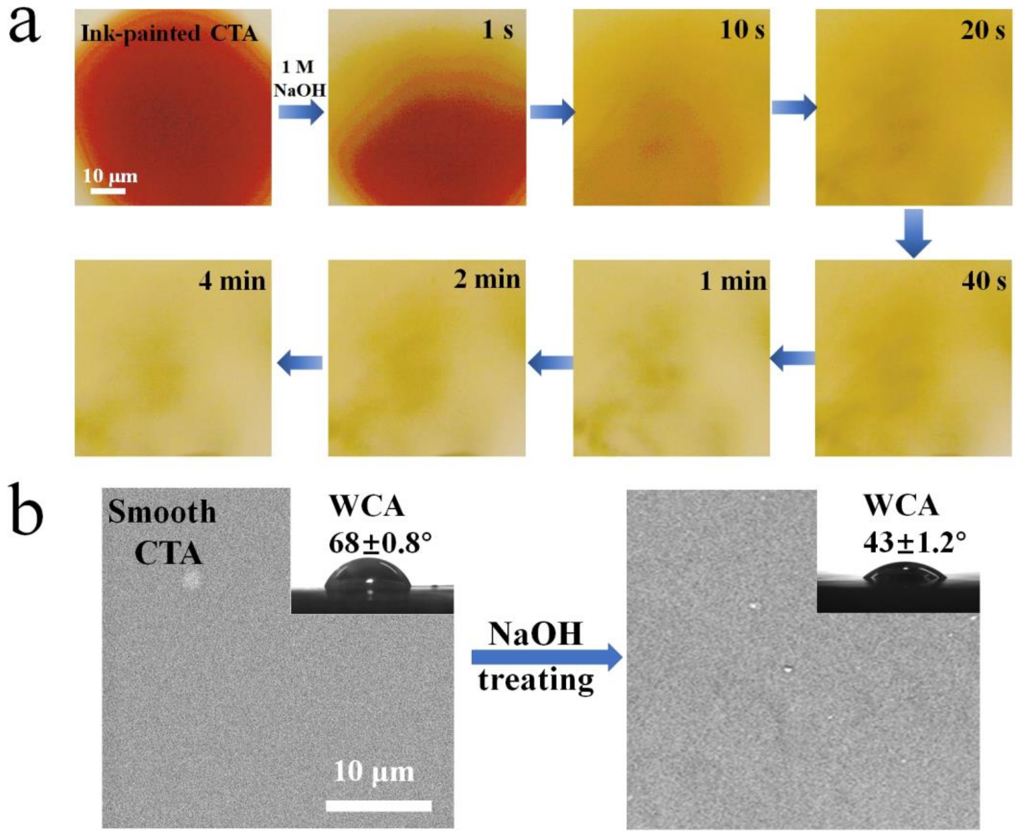

Supplementary Figure 7. NaOH etching of smooth CTA film. a Optical micrographs of the ink-painted smooth CTA surface during the dynamic etching process. **b** SEM images and water contact angles (WCAs) of the smooth CTA surface before and after treatment by 1M aqueous NaOH solution.

Supplementary Note 2. Effect of NaOH concentration on etching

Aqueous solutions with varied NaOH concentrations are used to treat the ink-painted HC. As shown in Supplementary Fig. 8, when the NaOH concentration is 0.1 M, it is too low to realize efficient etching, presenting an almost unchanged surface morphology even after 30-min treatment. When the concentration rises to 0.5 M, the top monolayer of 3D micropores disappears rapidly within 10 min, indicating a greatly improved etching rate. Further increasing the concentration to 2.0 M causes the critical etching time to decrease to 1 min. The results imply that the etching treatment, including the etching rate and critical etching time, can be adjusted by the NaOH concentration. Thereafter, a NaOH concentration of 1 M was selected for further exploration because of the suitable etching rate.

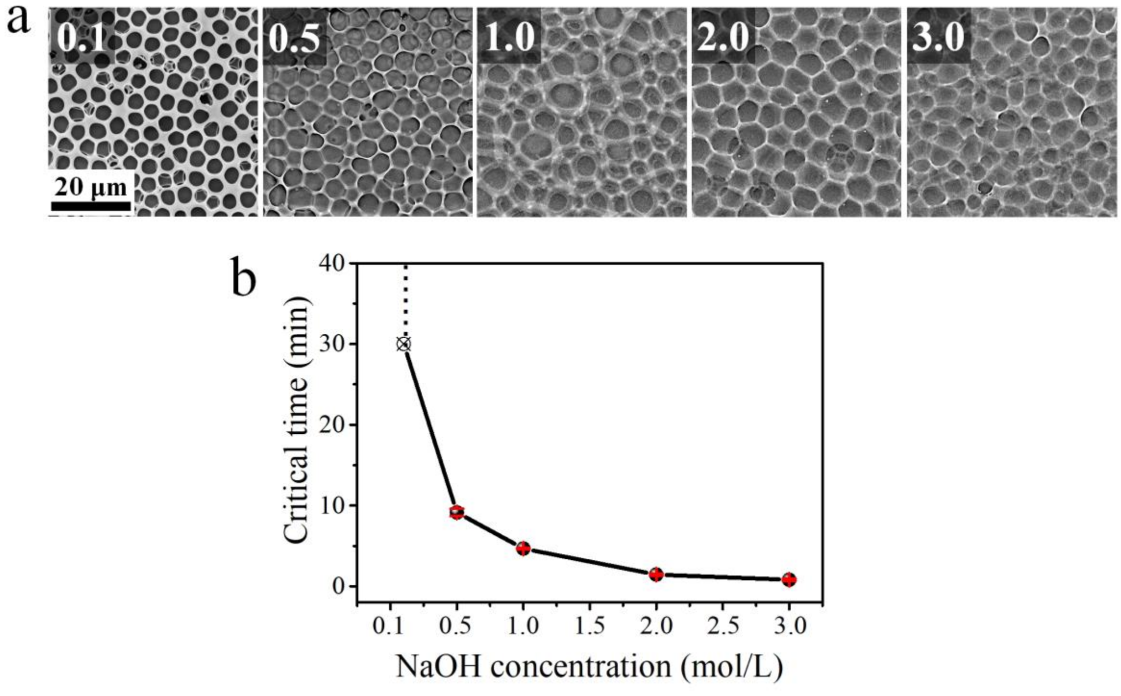

Supplementary Figure 8. Effect of NaOH concentration on etching. a Critical SEM images (except 0.1 M) of the ink-painted HC surface after NaOH-etching with increasing NaOH concentrations. **b** Critical etching times of ink-painted HC with increasing NaOH concentrations. The error bars in **b** represent standard deviations (n=3). Source data are provided as a Source Data file.

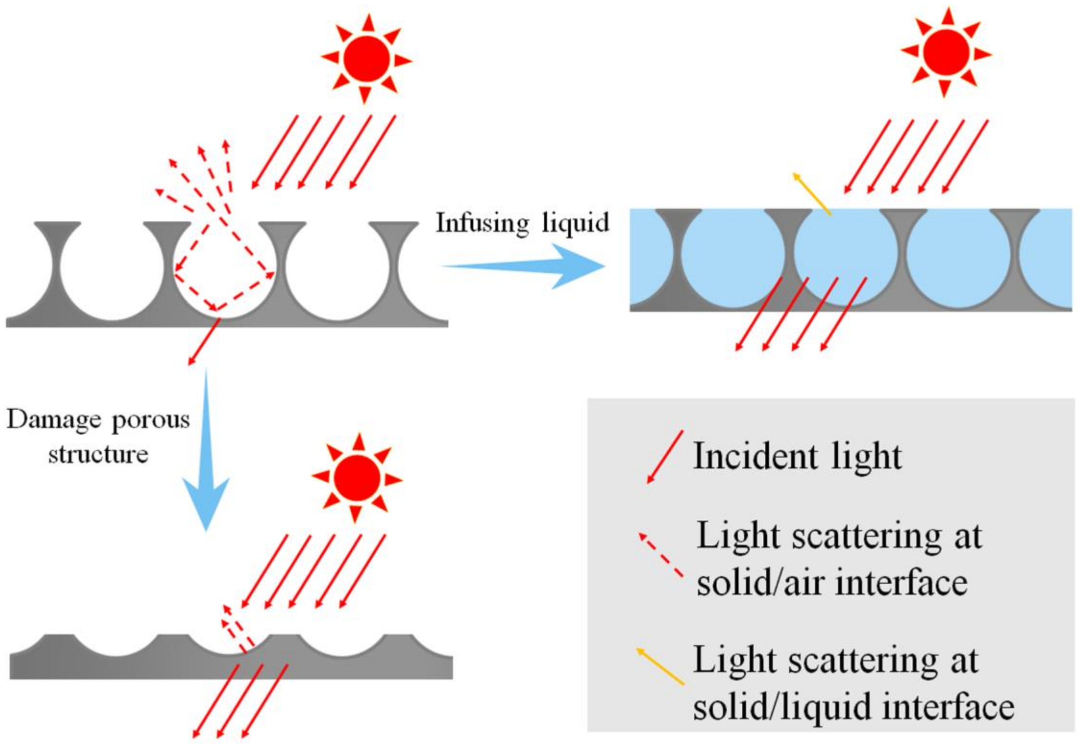

Supplementary Figure 9. Schematic illustration of transparency change for HC film. Strong scattering of light occurs in the presence of micropores, resulting in opacity. When the porous structures are liquid-infused or damaged, the light scattering is weakened, resulting in increasing transparency.

Supplementary Note 3. Effect of drying of ink on NaOH etching

Because the ink contains water, polyols and other volatile solvents, its drying is time-dependent; this possibly affects the further NaOH etching. To disclose the effect, the freshly prepared ink-painted samples were stored at 25℃ for different periods, and their WCAs as well as the dynamic etching process (1 M NaOH) were recorded. As shown in Supplementary Figure 10, the ink-painted HC surface is stable with nearly unchanged WCA (~28°) and critical etching time (~5 min) within 60-min storage. According to the optical micrograph of the surfaces in Supplementary Figure 11, it can be seen that the color saturation of the ink, before etching, remains unchanged, and the etching processes of the samples (within 60-min storage) are almost consistent. In contrast, with increases in the storage time to 10 h and even 1 d, despite the slightly increased WCA, the critical etching time still keeps approximately at 5 min (Supplementary Figures 12 and 13). Further extending the storage time to 5 d causes an obviously increased WCA (to ~57°) as well as critical etching time (to ~8 min). Moreover, if the freshly prepared ink-painted HC is dried at 100℃ for 2 h to remove the volatile solvents, its WCA and critical etching time increase to 66° and 10 min, respectively (Supplementary Fig. 14). Therefore, it can be concluded that the drying state of the ink-painted patterns only affects the surface wettability and etching behavior (critical time), and the etching can be also conducted normally. Considering the preparation efficiency and reliability, the etching treatment is conducted constantly within 1-h storage for evaluation.

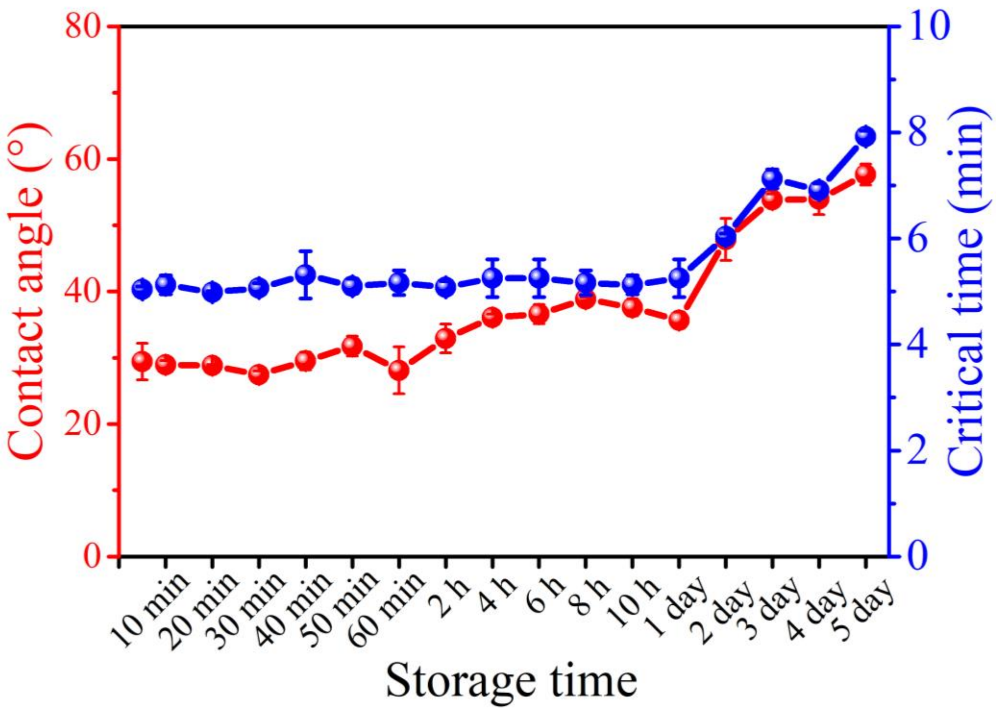

Supplementary Figure 10. Effect of storage time on NaOH etching. WCAs and critical etching times of ink-painted HC after storage at 25°C for different time periods. Error bars represent standard deviations (n=3). Source data are provided as a Source Data file.

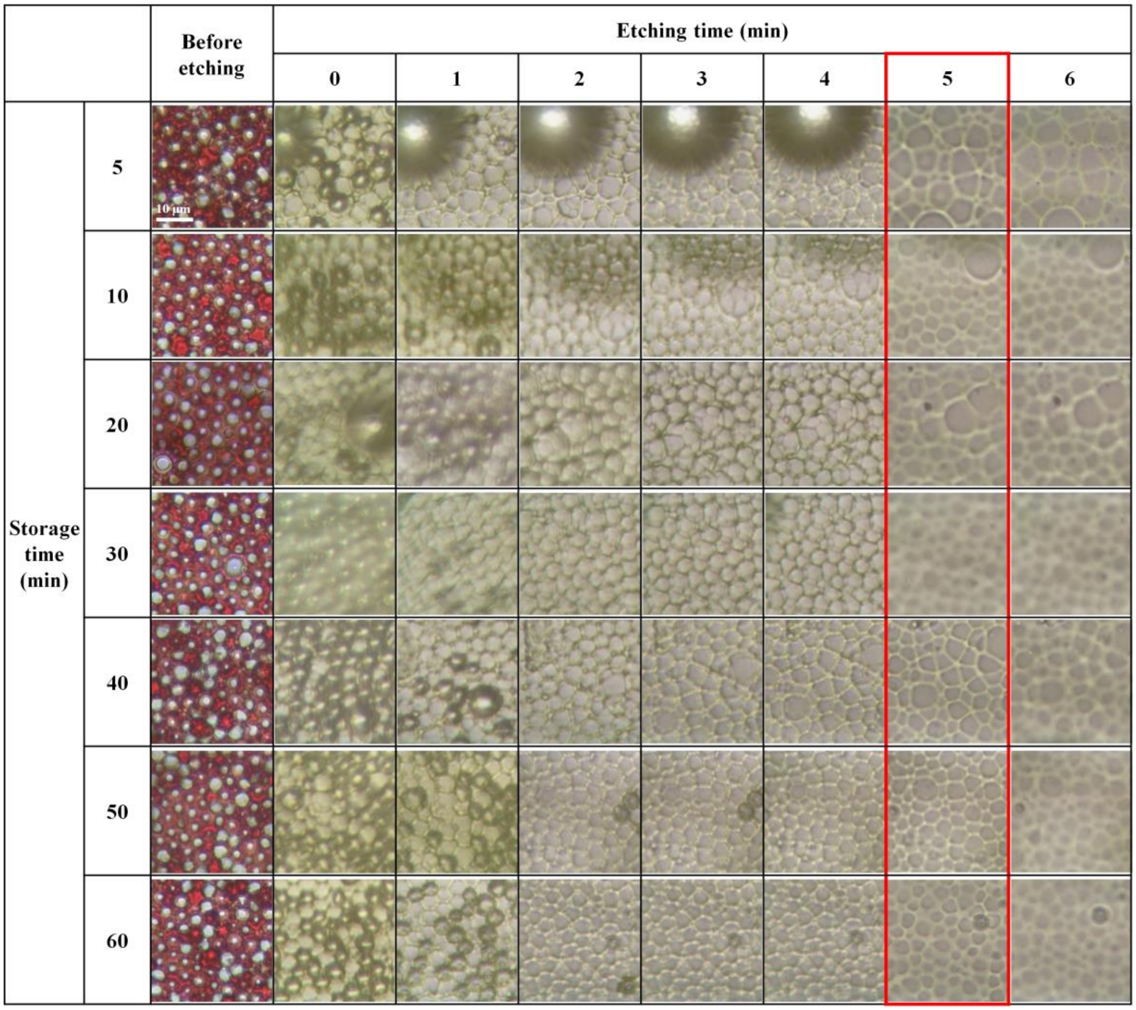

Supplementary Figure 11. Effect of ink drying within 60 minutes. Optical micrograph and dynamic etching process (1 M NaOH aqueous solution) of ink-painted HC in the case of within 60-min storage at 25℃. The critical state is marked with a red box.

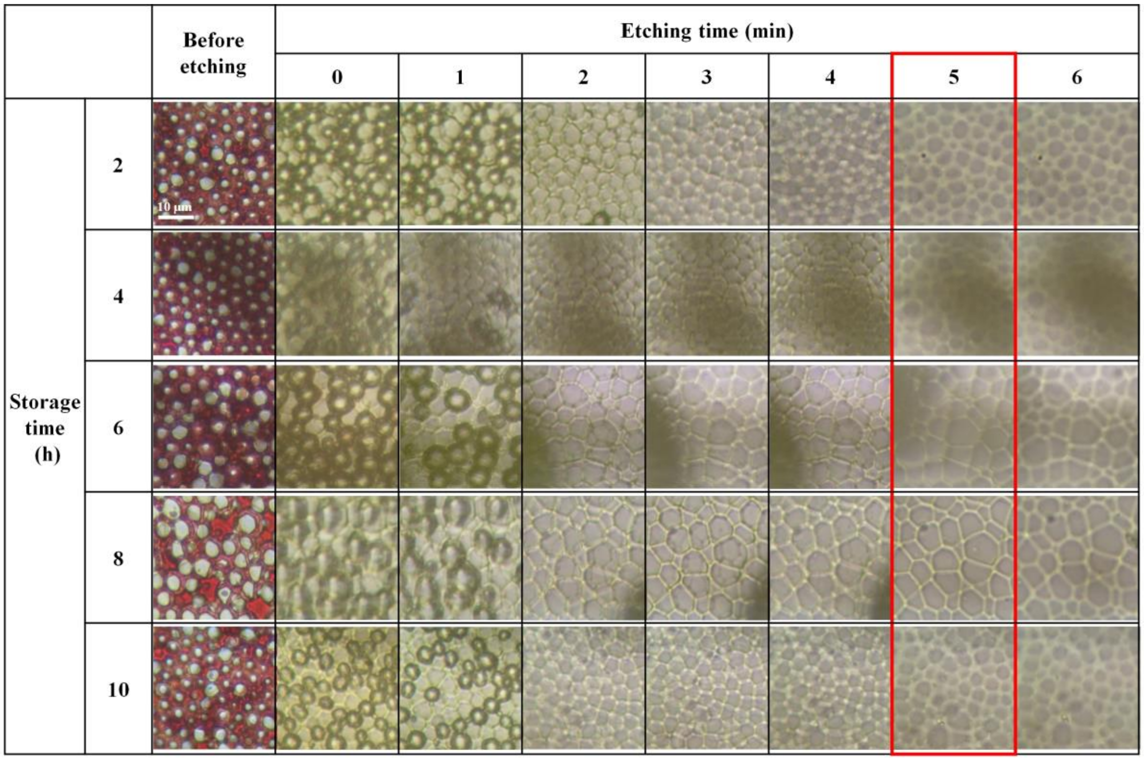

Supplementary Figure 12. Effect of ink drying within 10 hours. Optical micrograph and dynamic etching process (1 M NaOH aqueous solution) of ink-painted HC in the case of within 10-h storage at 25℃. The critical state is marked with a red box.

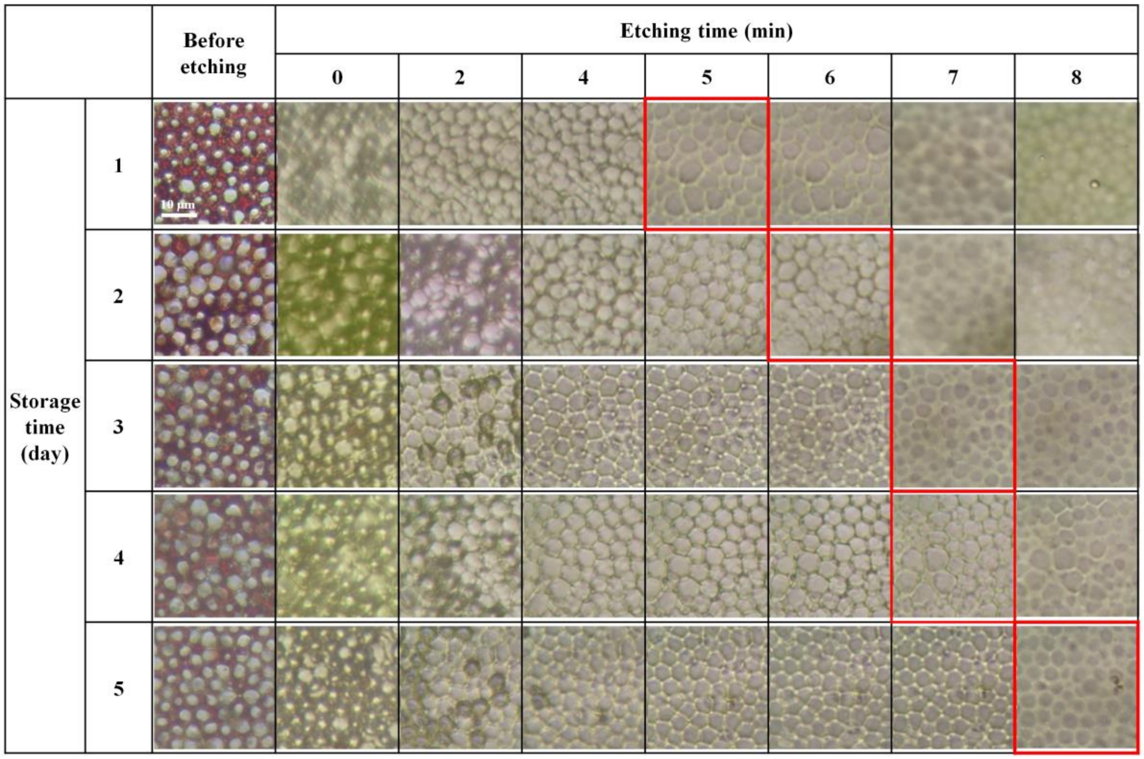

Supplementary Figure 13. Effect of ink drying within 5 days. Optical micrograph and dynamic etching process (1 M NaOH aqueous solution) of ink-painted HC in the case of within 5-d storage at 25℃. The critical state is marked with a red box.

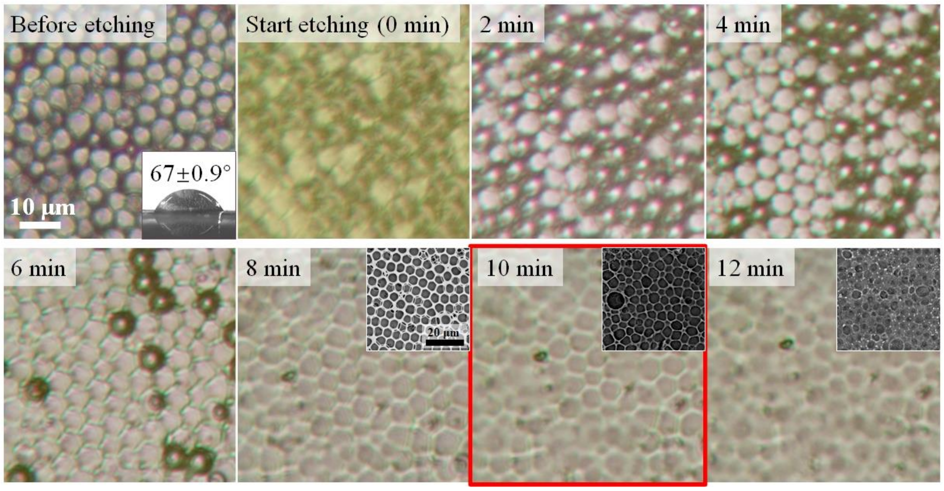

Supplementary Figure 14. Effect of high-temperature drying of ink on NaOH etching. Water contact angle and dynamic etching process (1 M NaOH aqueous solution) of ink-painted HC dried at 100℃ for 2 h. The critical state is marked with a red box.

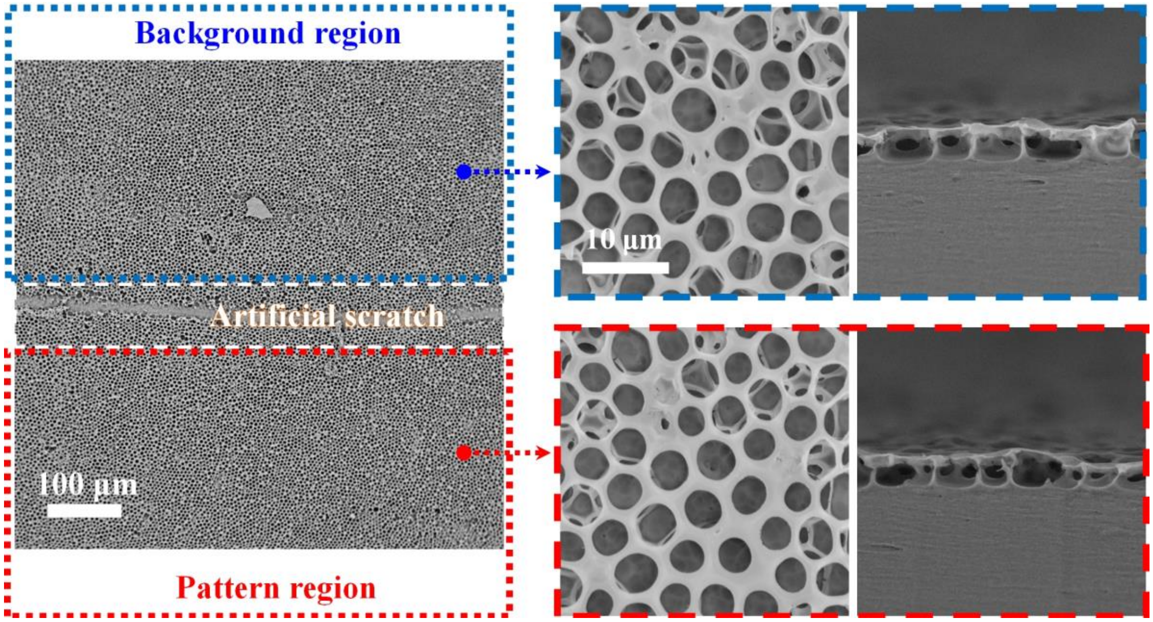

Supplementary Figure 15. Surface morphology of hidden-pattern HC surface. SEM images of background and pattern regions of a patterned HC surface prepared by 4-min etching with a 1 M NaOH aqueous solution. The scratch on the surface is artificially made to distinguish the boundary between the background region and pattern region.

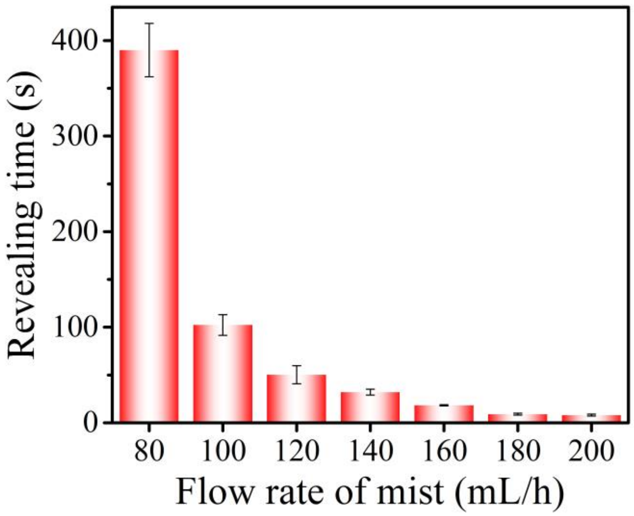

Supplementary Figure 16. Effect of mist flow rate on pattern revealing. Dependence of revealing time of hidden information on flow rate of mist. Error bars represent standard deviations (n=3). Source data are provided as a Source Data file.

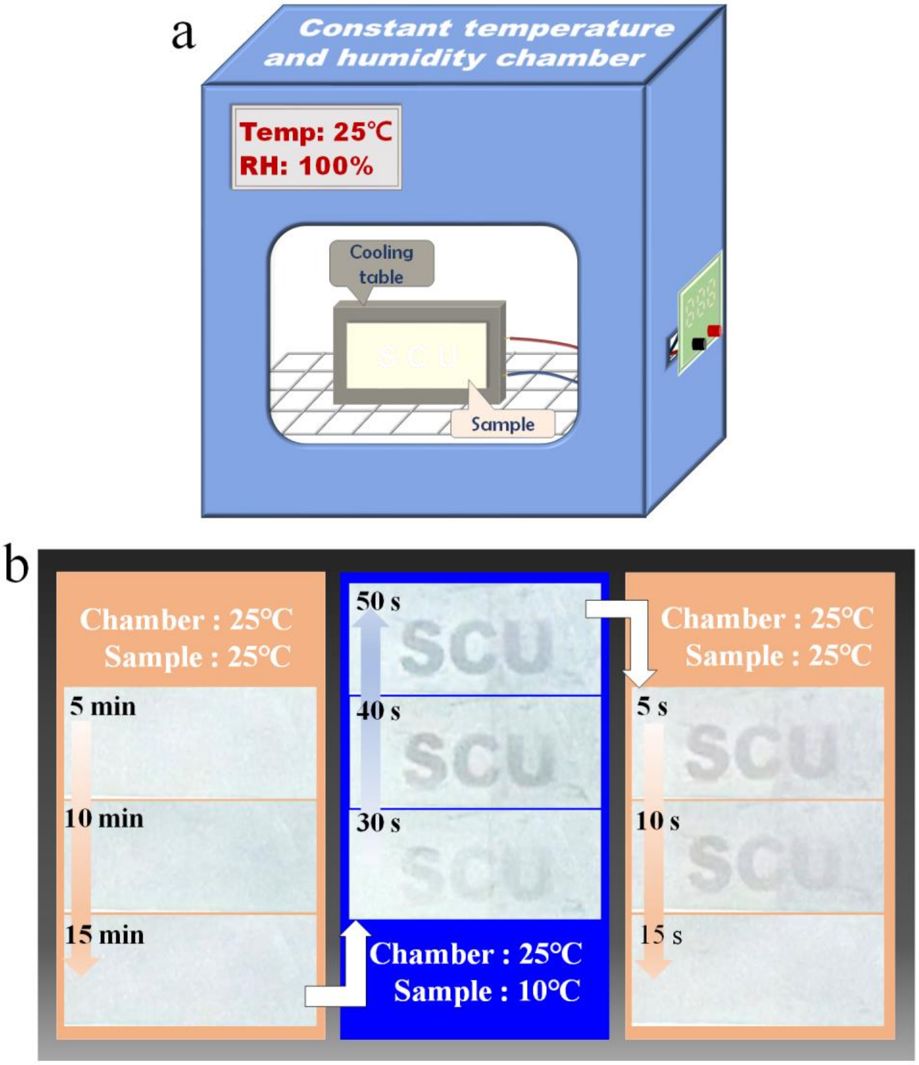

Supplementary Figure 17. Effect of humidity on pattern revealing. a Illustration of the test system for determining humidity stability. **b** Dynamic pattern changes upon controlling the sample temperatures at 100% RH.

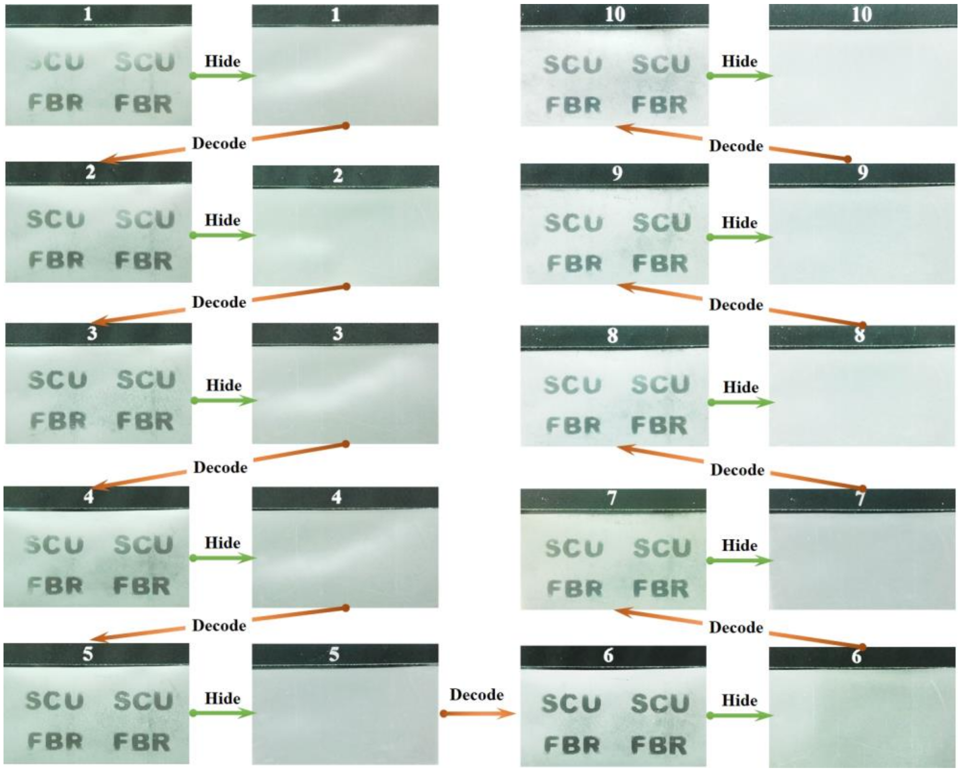

Supplementary Figure 18. Cyclic water-responsive revealing/hiding performances of patterns. The hiding/revealing behavior can be well repeated during ten cycles test.

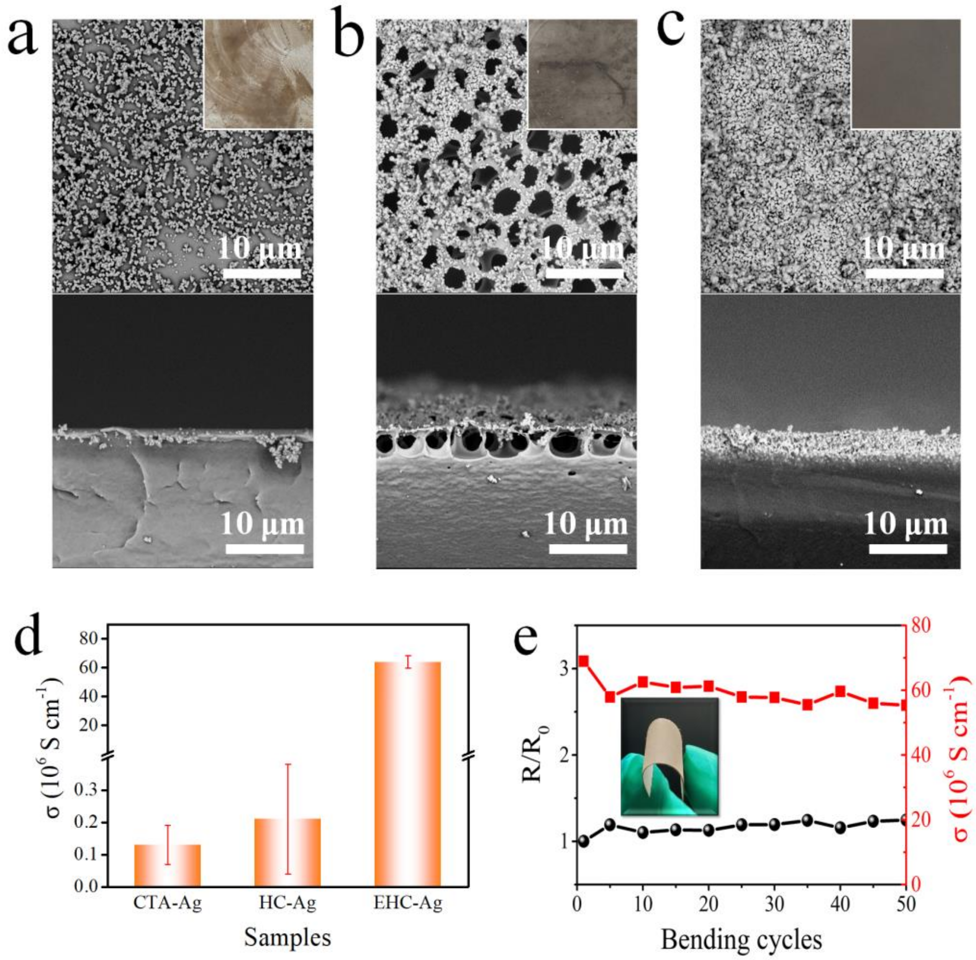

Supplementary Figure 19. Effect of surface morphology on conductivity. Digital images and SEM images of CTA-Ag (**a**), HC-Ag (**b**) and EHC-Ag (**c**). **d** Electrical conductivity of the CTA-Ag, HC-Ag and EHC-Ag surfaces. **e** Relative resistance and conductivity of the EHC-Ag surface during 50 repeated bending cycles. The error bars in **d** represent standard deviations (n=5). Source data are provided as a Source Data file.

Supplementary Tables

Supplementary Table 1. Summary of the surface wettability and morphology for different samples after being treated with 1 M NaOH aqueous solution for 0-10 min

Original sample	Etch time (min)	Wettability	Morphology	Dry state (in air)	Wet state (under water)
HC	0~10 min	Non-wettable	3D honeycomb-like porous surface	opaque	opaque
Ink-painted HC	0~2 min				
	3~4 min	wettable	3D honeycomb-like porous surface	opaque	transparent
	5~10 min	wettable	Etched porous surface	transparent	transparent

Supplementary methods

Revealing measurement of hidden patterns

For revealing measurements, the as-prepared samples were fixed on a holder with a setting distance of 10 cm between the sample and water mist outlet. Water mist was generated by using an ultrasonic humidifier (YC-E350, YADU, China). The flow rate was controlled within 80-200 mL/h. The revelation time of hidden patterns is recorded. The sample should be dried at 60℃ for pattern recovery before the following tests.

High-humidity revealing measurement was conducted by sticking samples on a semiconductor refrigeration platform with thermally conductive adhesive tape (Supplementary Figure 14a). The measurement was performed in a temperature and humidity test chamber (LHS-50CL, Yiheng, China), and changes of hidden patterns during the heating and cooling processes were recorded.
