# Supplementary material for: A confined-etching strategy for intrinsic anisotropic surface wetting patterning
Source: Nat Commun. 2022 Jun 2;13:3078. doi: 10.1038/s41467-022-30832-4 (PMC9163165; doi:10.1038/s41467-022-30832-4)
Supplement: Supplementary file 2 — Description of Additional Supplementary Files [file 41467_2022_30832_MOESM2_ESM.pdf]

Description of Additional Supplementary Files

Supplementary Movie 1

Legend: Sliding of water droplet on HC film. The methylene blue-stained water droplet can freely move on the HC surface without residue.

Supplementary Movie 2

Legend: Dynamic etching process of ink-painted HC. An ink-painted HC film was fixed onto glass slides, and 10 μL aqueous NaOH solution (1 M) was dripped onto the ink-painted surface. The surface morphological changes were observed under an optical microscope; the focal length of the microscope was fine-tuned for focusing images during the dynamic process.

Supplementary Movie 3

Legend: Water response of hidden patterns. Upon contact with water, the hidden pattern "FBR SCU" on the HC surface became visible immediately, showing a fast water response.

Supplementary Movie 4

Legend: Reversible decryption of hidden information. The hidden information of "FBR SCU" was decrypted by water mist. Accompanying natural drying of the surface, the revealed information recovered to the hidden state, which can be revealed again after a next water mist-treatment run.

Supplementary Movie 5

Legend: Stability of the hidden patterns in high-humidity environment. The high stability of hidden pattern was represented through 15-min storage at a constant 100% RH and 25°C. After cooling the film to 10°C, the hidden information was revealed quickly as a result of condensations of vaporous water into liquid water. Once the cooling stops, the visible patterns rapidly became invisible again.

Supplementary Movie 6

Legend: Reading process of hidden QR code by mobile phone. A QR code was successfully hidden with the HC surface which was unable to be read in air but appeared quickly underwater and could be read accurately.

Supplementary Movie 7

Legend: Stability of the distorted Ag electrode. The lamp in the conductive loop could work well during the cyclic bending-unbending operation of the Ag electrode.
